# Supplementary material for: MetaRibo-Seq measures translation in microbiomes
Source: Nat Commun. 2020 Jun 29;11:3268. doi: 10.1038/s41467-020-17081-z (PMC7324362; doi:10.1038/s41467-020-17081-z)
Supplement: Supplementary file 10 — Supplementary Data 7 [file 41467_2020_17081_MOESM10_ESM.zip › File2/Confidence_High_Taxonomy/43158_out.krona.html]

Javascript must be enabled to view this page.

members
magnitude
magnitudeUnassigned
count
unassigned
taxon
rank

43158\_out

2

superkingdom
2
2

2
phylum
1239

186801
class
2

order
186802
2

2
186803
family

genus
189330
2

species
1262873
2

SRS053214\_contig\_number\_contig-100\_5179.5180SRS148721\_contig\_number\_35937
